# Biophysical characterization of Cyclophilin B reveals membrane localization as its primary functional determinant as a prolyl isomerase

**DOI:** 10.1002/pro.70579

**Published:** 2026-04-16

**Authors:** Sarah C. DeVoe, Thomas C. Yost, Ashley J. Newton, Gavin A. Grever, Melissa Fernandez Ayala, Wendell P. Griffith, Robert D. Latvala, Philipp A. M. Schmidpeter

**Affiliations:** ^1^ Department of Chemistry The University of Texas at San Antonio San Antonio Texas USA

**Keywords:** Cyclophilin B, endoplasmic reticulum, peptidyl‐prolyl isomerase, protein folding, proteostasis, subcellular localization

## Abstract

The endoplasmic reticulum (ER) provides a specialized environment for the folding of secreted and membrane proteins, a process supported by many different chaperones. Among these chaperones, peptidyl‐prolyl *cis*/*trans* isomerases (PPIases) catalyze a rate‐limiting conformational step in protein folding, yet the principles governing isoform‐specific function of PPIases remain poorly defined. Cyclophilin B (CypB), an ER‐resident PPIase, has been implicated in early folding events, but whether its activity reflects biochemical adaptation to the ER environment is unclear. Here, we report the biophysical characterization of human CypB and compare it with the cytosolic isoform Cyclophilin A. Spectroscopic and enzymatic analyses show that CypB adopts the canonical cyclophilin fold and displays catalytic activity toward multiple substrates under both cytosolic‐ and ER‐mimicking conditions, indicating that its enzymatic properties are not uniquely tuned to the ER milieu. Confocal imaging confirms that full‐length CypB is enriched in the ER, and that removal of its N‐terminal segment disrupts this localization. Together, these results indicate that subcellular localization, mediated by an N‐terminal membrane anchor, rather than catalytic specialization, may define the physiological role of CypB. Our findings underscore compartmentalization as a central organizing principle of proteostasis in the secretory pathway.

## 1 INTRODUCTION

Approximately one‐third of all proteins in eukaryotic cells fold within the endoplasmic reticulum (ER), a specialized cellular compartment equipped with a dedicated set of chaperones that assist in protein folding and maintain protein quality control (Braakman and Hebert 2013; Brodsky and Skach 2011). Among these chaperones, peptidyl‐prolyl *cis*/*trans* isomerases (prolyl isomerases, PPIases) play a crucial role by catalyzing the *cis* ⇌ *trans* isomerization of peptidyl‐prolyl bonds (prolyl isomerization), a reaction that is inherently slow due to its high activation energy (~80 kJ mol^−1^) and often acts as a rate‐limiting step in protein folding (Brandts et al. 1975; Schonbrunner and Schmid 1992; Schmid et al. 1993; Pradeep et al. 2006; Schmidpeter and Schmid 2015a, 2015b). Beyond protein folding, prolyl isomerization can serve as a molecular switch or timer, regulating protein activity and stability (Andreotti 2003; Breheny et al. 2003; Eckert et al. 2005; Lu et al. 2007; Rognoni et al. 2014; Sarkar et al. 2007; Schmidpeter et al. 2020; Schmidpeter and Schmid 2015a, 2015b; Shinoda and Fujitani 2017). To prevent the accumulation of aggregation‐prone folding intermediates due to incorrect proline isomers, cells universally express three main families of PPIases (Gothel and Marahiel 1999): cyclophilins (Cyp), FK506‐binding proteins (FKBP), and parvulins (Par). The activity of each family largely depends on the amino acid residue preceding proline, and the presence of chaperone domains fused to the catalytic PPIase domain can markedly enhance enzyme–substrate interactions (Jakob et al. 2009; Janowski et al. 1997; Zoldak et al. 2009). These features highlight the role of PPIases in maintaining proteostasis and emphasize their importance in cellular function and protein stability.

Interestingly, only a small number of PPIases are present in the ER, despite the central role of this compartment in protein folding (Boudko et al. 2014; Stocki et al. 2014). While detailed studies of PPIase function have been carried out for cytosolic isoforms, data on their organelle‐specific homologs are limited (Davis et al. 2010; Jurkova et al. 2025). Given the essential role of the ER in protein folding, it is important to characterize the functions of ER‐resident chaperones and describe their adaptation to specific chemical environments.

**FIGURE 1:**
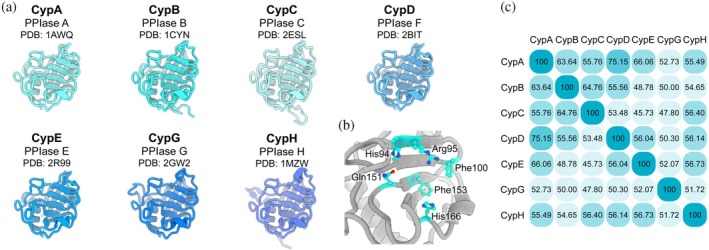
Structure and sequence conservation across cyclophilins. (a) Structures of human cyclophilins in ribbon representation. Names (Cyp), alternative names (PPIase), and PDB identifiers are given. (b) Zoom of the catalytic site of Cyclophilin B (PDB: 1CYN) with catalytically important residues in ball‐and‐stick representation. (c) Sequence conservation among cyclophilins generated using CLUSTAL O (1.2.4) with the UniProt entries for human cyclophilins: P62937 (CypA), P23284 (CypB), P45877 (CypC), P30405 (CypD), Q9UNP9 (CypE), Q13427 (CypG), and O43447 (CypH).

Cyclophilins are a family of PPIases involved in various functions across cellular compartments, including protein folding and trafficking, cell signaling, apoptosis, gene expression, and viral replication (Davis et al. 2010; Marks 1996), and, accordingly, are enticing drug targets (Handschumacher et al. 1984; Jurkova et al. 2025; Peterson et al. 2022; Stauffer et al. 2024). Cyclophilin A was initially discovered because of its high affinity for the immunosuppressive drug cyclosporin A and has since been recognized as the primary cytosolic cyclophilin (Fischer et al. 1989; Handschumacher et al. 1984; Takahashi et al. 1989). In cellular organelles, distinct cyclophilins have been identified, including Cyclophilin B (CypB) in the ER lumen and Cyclophilin D (CypD) in the mitochondrial matrix (Price et al. 1991). These enzymes share high sequence similarity with CypA, with the main differences being the N‐terminal peptides that target CypB and CypD to specific cellular compartments (Figures 1, S1, and S2a,b) (Price et al. 1991). Cyclophilins can be single‐domain enzymes composed of only the catalytic domain or multi‐domain enzymes where the catalytic domain is fused to other functional domains that facilitate protein–protein interactions (Davis et al. 2010; Schiene‐Fischer 2015). The core structure of these proteins consists of two antiparallel β‐sheets flanked by two short α‐helices (Figure 1a). This conserved structure contains the active‐site residues essential for PPIase activity (Figures 1b and S1). CypA exhibits high catalytic activity, indicated by the specificity constant *k*_cat_/*K*_M_, with minimal preference for the amino acids flanking proline (Janowski et al. 1997; Schmidpeter et al. 2011; Zoldak et al. 2009).

Cyclophilin B has been associated with collagen folding and maturation, can act as a proinflammatory factor, and participates in hormone signaling, among other functions (Gegunde et al. 2021; Rycyzyn et al. 2000; Terajima et al. 2019). The main difference to CypA is a putative N‐terminal transmembrane helix, which targets CypB to the ER lumen and the secretory pathway (Figures 1c, S1, and S2a,b) (Arber et al. 1992; Bergsma et al. 1991; Price et al. 1991; Price et al. 1994; Tanveer et al. 1996). In the ER, CypB likely helps fold secreted and membrane proteins and is a central contributor to cellular proteostasis and protein quality control (Braakman and Hebert 2013; Jeong et al. 2015; Kim et al. 2008; Kozlov et al. 2010; Kozlov and Gehring 2020; Stocki et al. 2014). This requires CypB to be active in the ER lumen, an environment characterized by high calcium levels and an oxidative redox potential. These conditions are unique to the ER and may influence CypB PPIase activity. However, despite its physiological importance, a comprehensive biophysical characterization of CypB is lacking, leaving critical gaps in understanding its role in ER protein homeostasis.

Here, we report the detailed biophysical characterization of human Cyclophilin B. Using spectroscopic experiments, we investigated the structural integrity, conformational stability, and prolyl isomerase activity of CypB and compared our results with those for the well‐characterized PPIase CypA. These analyses reveal that the catalytic activity of CypB is not specifically adapted to the chemical environment of the ER. Instead, the N‐terminus of CypB, which is predicted to form a transmembrane helix, emerges as a key factor differentiating the physiological function of CypB from CypA. Our results suggest that cellular localization, rather than catalytic divergence, may be the primary determinant of physiological specificity for cyclophilins.

## 2 RESULTS

Structural data for human cyclophilins are available from X‐ray crystallography and solution NMR studies, showing the conserved fold of the catalytic domain (Figure 1a). Together with the high sequence conservation across all isoforms (Figures 1c and S1), it is essential to describe the functions of the different cyclophilin isoforms to better understand their individual physiological roles. For example, CypB is presumably located in the endoplasmic reticulum (ER) (Arber et al. 1992; Price et al. 1991), indicating an important role for this enzyme in the folding of membrane proteins. However, detailed biophysical data on the stability and function of CypB remain limited (Kozlov et al. 2010; Mikol et al. 1994). Here, we studied the ER‐resident prolyl isomerase CypB and compared it with the well‐characterized cytosolic isoform CypA.

### 2.1 Structural integrity of Cyclophilin B

**FIGURE 2:**
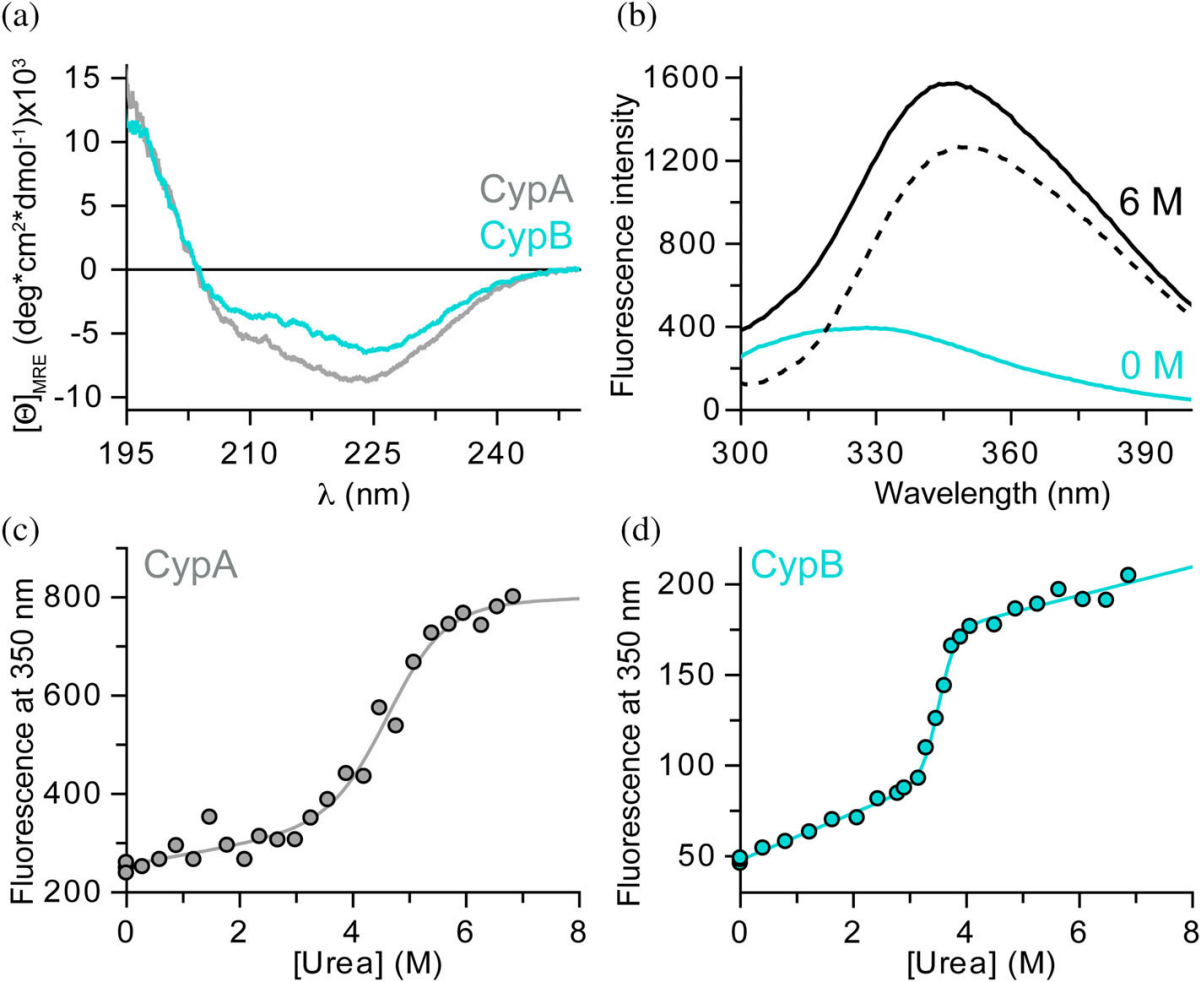
Folding and stability of Cyclophilin A and B. (a) Circular dichroism spectra are presented as mean residue ellipticity ([Θ]_MRE_, Equation (1)) for CypA (gray) and CypB (turquoise) measured from 250 to 195 nm (in 10 mM Na‐phosphate, pH 7.4, at 15 °C). (b) Fluorescence spectra of CypB with 0 M (turquoise) and 6 M urea (black line). The dashed line indicates the difference spectrum between 6 M and 0 M urea. Fluorescence was recorded after excitation at 280 nm (1 μM protein in 20 mM HEPES, 100 mM KCl, pH 7.4, at 15 °C). (c, d) Urea‐induced unfolding transitions of CypA (c) and CypB (d). Fluorescence signals (excitation: 280 nm, emission: 350 nm) of 1 μM protein were recorded (20 mM HEPES, 100 mM KCl, pH 7.4, with increasing concentrations of urea). Lines represent fits (Equation (2)) according to a two‐state model.

The *PPIB* gene encodes the catalytic cyclophilin domain and an N‐terminal helix that includes a putative signal peptide and is thought to interact with the membrane and protein‐binding partners (Figure S2a,b) (Arber et al. 1992; Price et al. 1991). This predicted transmembrane helix inhibits the expression of the full‐length protein in bacteria, and truncation of the first 28 amino acids was necessary to successfully express CypB in *E. coli* and obtain pure, homogeneous protein for in vitro experiments (Kozlov et al. 2010; Krogh et al. 2001; Mikol et al. 1994; Price et al. 1991). We will refer to this truncated construct as CypB and use this protein, unless otherwise noted, to compare its properties with those of the well‐studied CypA (Figure S2a–d). First, we confirmed the identities of purified CypA and CypB using high‐resolution mass spectrometry (Figure S2f,g). To ensure that the proteins used in this study are correctly folded, we recorded the far‐UV circular dichroism (CD) spectra of both (Figure 2a). The CD signal, which is sensitive to α‐helical and β‐sheet structures, reflects these features as expected from the known structures of cyclophilins (Figure 1a). The minimum at 222 nm indicates the presence of α‐helical segments, while the minimum at 208 nm is less pronounced due to overlapping signal from the pronounced β‐sheet around the catalytic core of cyclophilins (Figures 1 and 2a). Additionally, both spectra showed a distinct positive signal at wavelengths below 200 nm, indicating the presence of folded proteins. Furthermore, the normalized CD spectrum (mean residue ellipticity, [Θ]_MRE_; Equation (1)) of CypB closely resembles that of CypA, consistent with the highly conserved structure of the cyclophilin catalytic domain (Figures 1a and 2a) (Davis et al. 2010).

### 2.2 Conformational stability of CypB

To compare the conformational stability of CypA and CypB, we next performed urea‐induced equilibrium unfolding studies. The intrinsic protein fluorescence of both proteins (CypA: 1x Trp, 2x Tyr; CypB: 2x Trp, 4x Tyr) is well‐suited as a readout for the conformational folding of cyclophilins. It increases with increasing urea concentration, as evident in the fluorescence spectra at 0 M and 6 M urea, with a maximum signal change at 350 nm upon excitation at 280 nm (Figure 2b), providing a sensitive measure for analyzing the conformational stability of these proteins.

**FIGURE 3:**
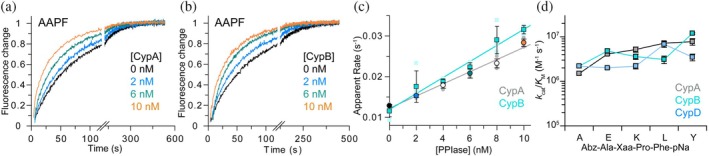
Catalytic activity of cyclophilins. (a, b) Normalized fluorescence recordings for the peptide Abz‐Ala‐Ala‐Pro‐Phe‐pNA with increasing PPIase concentration: 0 nM, black; 2 nM, blue; 6 nM, green; 10 nM, orange for CypA in (a), CypB in (b) (50 mM K_2_HPO_4_, 1 mM EDTA, pH 7.4, 1 mM DTT, at 15 °C). (c) Apparent rate of fluorescence change (which reports prolyl isomerization) from kinetics as shown in (a, b) as a function of the PPIase concentration. Data points are mean ± SD from at least three repeats. CypA with colors as in (a) and additional PPIase concentrations in gray, CypB in turquoise. Individual data points are in light colors. Lines are linear fits to averaged data points to determine *k*_cat_/*K*_M_ using Equation (1). (d) Catalytic activity (specificity constant, *k*_cat_/*K*_M_) from plots as in (c) for all five peptides used in this study and three cyclophilin isoforms (CypA, gray; CypB, turquoise; CypD, light blue). Error bars are SD from linear regression as shown in (c).

We incubated native proteins with increasing urea concentrations and measured the fluorescence intensity of each sample at 350 nm (excitation at 280 nm; Figure 3c,d). Both CypA and CypB exhibited cooperative unfolding transitions that were well described by a two‐state model of protein folding (N⇌U), which assumes only fully folded (*N*) and unfolded (*U*) molecules. Applying the linear extrapolation method (Equation (2)) (Santoro and Bolen 1988) allowed us to determine the midpoints of their transitions as 4.6 ± 0.3 M urea for CypA and 3.6 ± 0.1 M urea for CypB from three independent experiments (Figure 2c,d and Table S1). Together, our initial characterization shows that both cyclophilins central to this study are well‐folded when expressed and purified from bacterial systems, making them suitable for functional characterization of their catalytic activities.

### 2.3 CypB PPIase activity is characteristic of cyclophilins

It has previously been demonstrated that CypB exhibits PPIase activity, but many details, such as sequence specificity and whether different PPIase isoforms have evolved for specific functions, remain unknown (Davis et al. 2010; Mikol et al. 1994). To address this, we next analyzed the prolyl isomerase activity of CypB using an established, protease‐free fluorescent peptide assay (Zoldak et al. 2009). This assay directly probes the catalytic activity of prolyl isomerases. Tetrapeptides with the general formula Abz‐Ala‐Xaa‐Pro‐Phe‐pNA (Abz = amino benzoyl, Xaa = any amino acid, pNA = para‐Nitroanilide) were solubilized in trifluoro ethanol (TFE) supplemented with 0.55 M anhydrous LiCl. Under these conditions, prolyl bonds predominantly adopt the *cis* conformation, placing the fluorophore (pNA) and fluorescence quencher (Abz) in close proximity, resulting in a low fluorescence signal. Upon transfer of the peptide to aqueous buffer, the *cis*/*trans* equilibrium is shifted toward the *trans*‐Pro species, and the re‐equilibration can be monitored by measuring the increase in pNA fluorescence (Zoldak et al. 2009) (Figure 3a,b).

We used five peptides with either Ala, Glu, Lys, Leu, or Tyr preceding proline, allowing us to evaluate the activity and sequence specificity of cyclophilins. To establish the accuracy of the assay in our hands, we first measured the activity of CypA. In cells, CypA is localized in the cytosol, a reducing environment. Therefore, we performed the peptide‐based isomerization assay under reducing conditions (1 mM Dithiothreitol, DTT). We varied the PPIase concentration from 0 to 10 nM, fitted the fluorescence time courses with an exponential function, and plotted the apparent rate of fluorescence increase as a function of PPIase concentration to determine the enzymatic activity (Figures 3a,c and S3a) (Equation (3)). In the absence of enzyme, the fluorescence increase was slow for all peptides, reflecting the rate of the uncatalyzed reaction (*k*_0_ in Equation (3)) (Figures 3a,b and S3a). In the presence of nanomolar concentrations of CypA, the measured fluorescence increase, which reports peptidyl‐prolyl *cis*/*trans* isomerization, was faster than the uncatalyzed reaction. Furthermore, the isomerization rate increased linearly with enzyme concentration (Figures 3a,c and S3a), allowing us to determine the catalytic efficiency (*k*_cat_/*K*_M_) using Equation (3).

As previously reported, the well‐characterized, cytosolic cyclophilin, CypA, exhibits high catalytic efficiency with *k*_cat_/*K*_M_ values ranging from 10^6^ to 10^7^ M^−1^ s^−1^, without a clear preference for the amino acid preceding proline (Schmidpeter et al. 2011; Zoldak et al. 2009) (Figure 3d). To further validate the reliability of the peptide‐based PPIase assay, we performed independent control experiments using two additional PPIases: mitochondrial Cyclophilin D (CypD), which shares a high sequence similarity to CypA (Figures 1c and S1), and the bacterial FKBP‐type PPIase SlyD (Scholz et al. 2006; Zoldak et al. 2009). Across all five peptides tested, CypD exhibits the same high activity as CypA, without noticeable sequence specificity for the residue preceding the proline (Figures 3d and S3c and Table S2). Unlike cyclophilins, FKBP‐type PPIases show pronounced sequence specificity and strongly favor hydrophobic amino acids over anionic ones at the position preceding proline (Schmidpeter et al. 2011; Zoldak et al. 2009). Testing the catalytic efficiency of SlyD for two peptides (ALPF and AEPF; Figure S3d) recapitulated this reported behavior. Together, our data for CypA, CypD, and SlyD validate the sensitivity, specificity, and accuracy of the peptide‐based isomerization assay.

Next, we measured the activity of CypB in the peptide‐based isomerization assay and observed the same high activity as for CypA and CypD. Across all five peptides, the determined *k*_cat_/*K*_M_ values range from 10^6^ to 10^7^ M^−1^ s^−1^ (Figures 3b–d and S3b and Table S2), demonstrating that high catalytic activity without preference for the amino acid preceding proline is conserved among CypA, CypB, and CypD. This also shows that CypB is well‐suited to assist in a wide range of protein folding reactions.

### 2.4 PPIase activity under ER‐mimicking conditions

CypB is believed to be localized to the ER (Arber et al. 1992; Price et al. 1991), which is characterized by its oxidative environment and high Ca^2+^ concentrations. These features are necessary for the primary functions of the ER, including Ca^2+^ storage, lipid biosynthesis, membrane protein folding, and oxidative protein folding (Braakman and Hebert 2013). To test whether the PPIase activity of CypB is specifically adapted to these unique conditions, we next performed the peptide‐based isomerization assay in the presence of 2 mM Ca^2+^ under oxidizing conditions (5 mM glutathione disulfide, GSSG; 1 mM glutathione, GSH).

**FIGURE 4:**
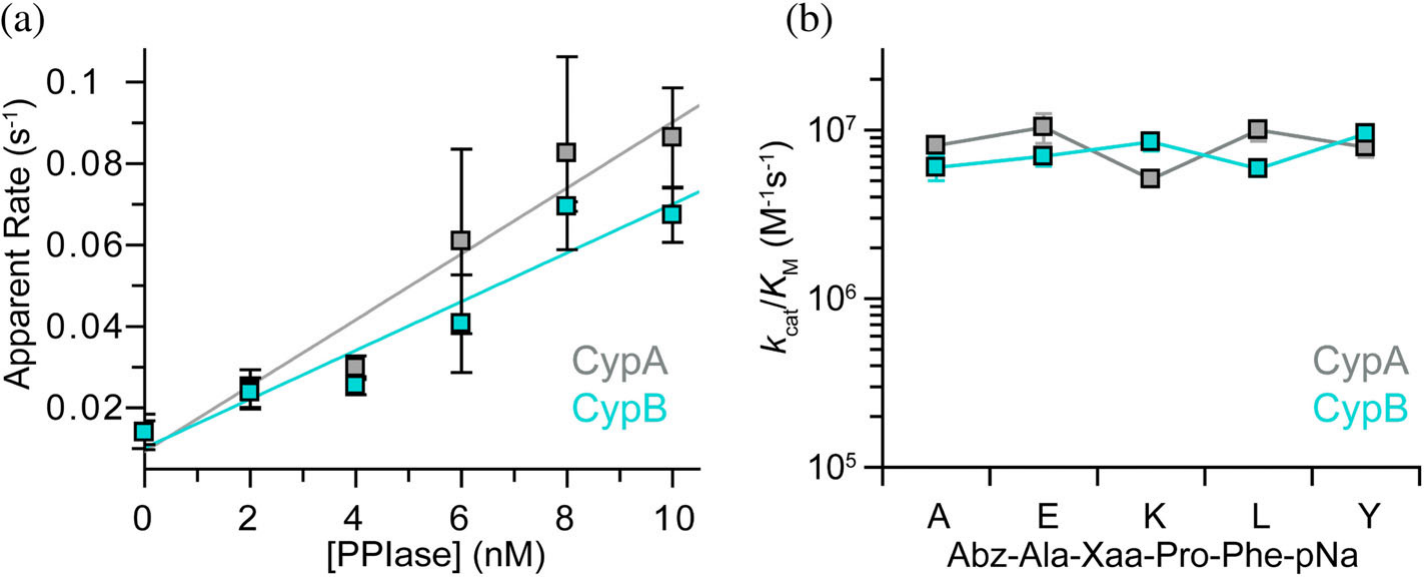
Catalytic activity of cyclophilins under ER‐mimicking conditions. (a) Rate of the observed fluorescence change (which reports on prolyl isomerization) as a function of the PPIase concentration (CypA, gray; CypB, turquoise) of the tetrapeptide Abz‐Ala‐Ala‐Pro‐Phe‐pNA in 20 mM HEPES, 100 mM KCl, 2 mM CaCl_2_, pH 7.4, 1 mM GSH, 5 mM GSSG, at 15°C. Error bars are SD from at least three repeats. (b) Catalytic activity (*k*_cat_/*K*_M_) of CypA (gray) and CypB (turquoise) under ER‐mimicking conditions from plots as in (a) for the five peptides used in this study, as indicated along the x‐axis. Error bars are SD from linear regression as shown in (a).

Across all five peptides, we observed high activity of CypB without noticeable preferences for the chemical nature of the amino acid preceding proline (Figure 4, turquoise data points; Figure S4 and Table S3). Despite the high sequence similarity between CypA and CypB (Figure 1c), there are sufficient amino acid differences that may have evolved for the optimized function of CypB in the ER (Figure S1). To test for this possibility, we next measured the activity of CypA under ER‐mimicking conditions. Similar to CypB and our results in reducing environments (Figure 3), CypA showed high catalytic activity across all peptide substrates (Figure 4, gray data points; Figure S4 and Table S3). This demonstrates that cyclophilins maintain high catalytic activity across various cellular conditions, such as the reducing cytosol and the oxidizing ER lumen, suggesting that different isoforms are not used to adapt the catalytic activity of these PPIases to distinct cellular environments.

### 2.5 Full‐length CypB is localized in the ER

**FIGURE 5:**
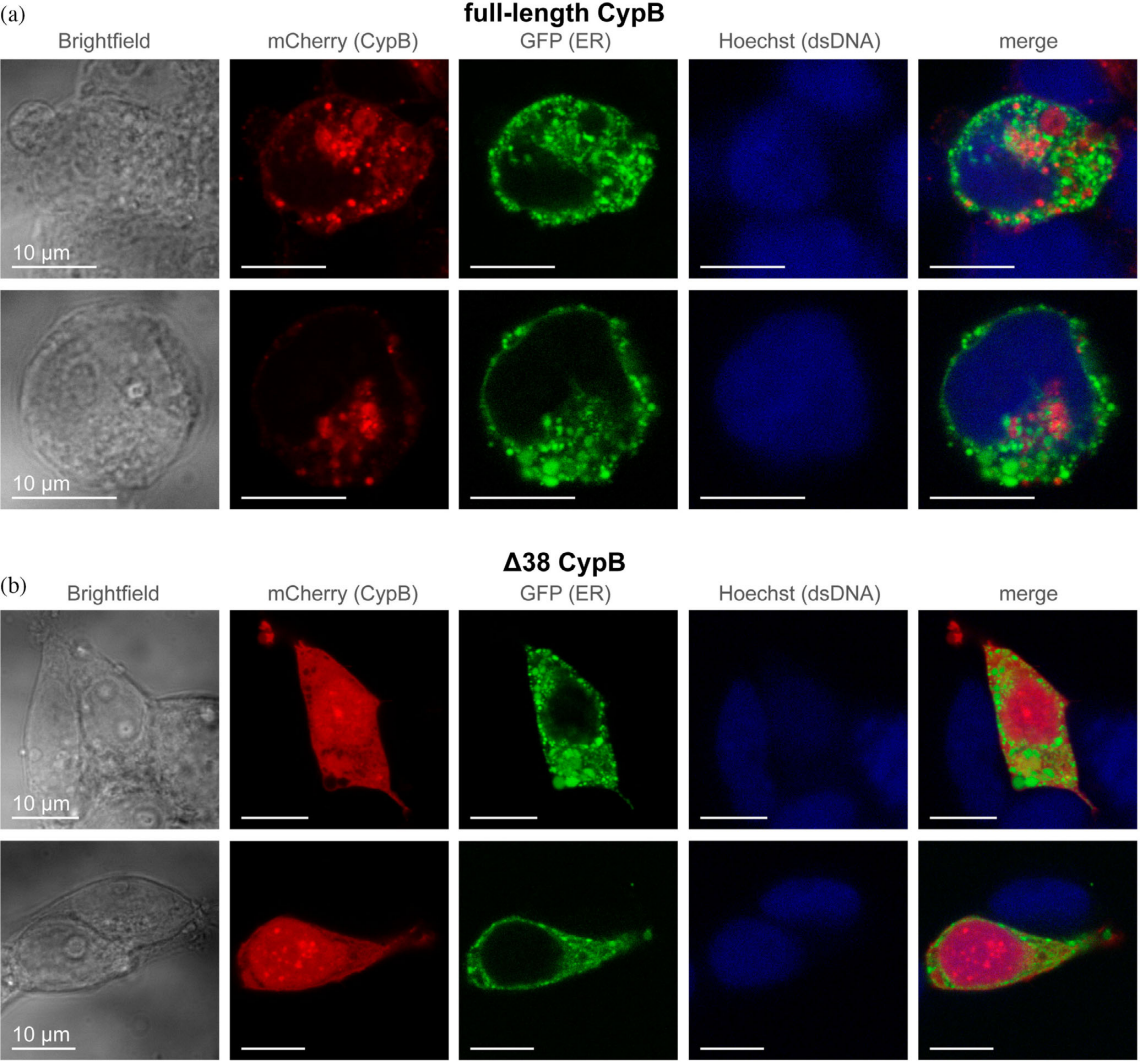
Cellular localization of CypB. (a, b) Confocal microscopy of HEK cells captured in the center slice of the cell. Columns are (from left to right): brightfield, mCherry (CypB), GFP (ER), Hoechst (dsDNA), merge of the three fluorescence channels. The CypB variants expressed are full‐length human CypB (in (a)), and human CypB with the first 38 amino acids deleted (Δ38 CypB, in (b)). The scale bar in all images is 10 μm.

Our functional analysis revealed that the catalytic domain of CypB is a highly active PPIase, and that its catalytic activity is independent of conditions typical for the ER. In cells, CypB is thought to be localized to the ER membrane via an N‐terminal helix (Figure S2a,b), which prevents the expression of full‐length CypB in bacteria (Davis et al. 2010; Kozlov et al. 2010; Mikol et al. 1994; Price et al. 1991). In contrast, full‐length CypB can be efficiently expressed in mammalian cells, such as human embryonic kidney cells (HEK 293S GnTi^−^), as demonstrated by confocal fluorescence microscopy of cells expressing the full‐length CypB‐mCherry fusion protein (Figure 5a) (Price et al. 1991). However, during purification from HEK cell cultures, full‐length CypB was partially degraded, and the experimentally determined mass of the final protein was ~3.6 kDa smaller than the calculated mass of the tag‐free construct. This mass difference can be explained only by truncations at both the N‐ and C‐termini, which is further supported by mass spectrometry‐based peptide mapping (Figure S5). This renders CypB purified from HEK cells unsuitable for studying the activity of the full‐length protein.

Nevertheless, in agreement with previous reports (Price et al. 1991; Price et al. 1994), confocal microscopy imaging confirmed that full‐length CypB‐mCherry is not evenly distributed throughout the cells, even under overexpression conditions. The mCherry signal (CypB) showed a strong correlation with ER‐specific staining (GFP signal in Figure 5). This confirms that full‐length CypB is efficiently tethered to the endomembrane system, likely the ER. Notably, despite the presence of the predicted N‐terminal transmembrane helix (Figure S2a,b), only minimal signal appears at the plasma membrane. The ER‐specific stain also efficiently detects ER‐plasma membrane contact sites (Figures 5a and S6a), leading us to conclude that, even when overexpressed, full‐length CypB is mainly localized to the early secretory pathway.

In contrast, when we expressed a truncated form of this enzyme lacking the first 38 amino acids in HEK cells (Δ38 CypB; Figure S2a), the protein (Δ38 CypB‐mCherry) was evenly distributed throughout the cell (Figures 5b and S6). This indicates that the predicted N‐terminal helix is necessary and sufficient to target CypB to the ER membrane. Combined with our in vitro functional results, which show that the PPIase activity of CypB is unchanged under cytosolic and ER‐mimicking conditions, this suggests that subcellular localization, driven by the N‐terminal helix, is a key determinant of CypB function as a protein folding helper enzyme in cells.

## 3 DISCUSSION

Protein folding is a crucial process in all living cells, relying on an array of chaperones to guide linear polypeptides into their proper, three‐dimensional structure. Many of these chaperones exist in multiple isoforms. For example, in humans, the cyclophilin family of peptidyl‐prolyl *cis*/*trans* isomerases includes seven members that all catalyze the same reaction (Bergsma et al. 1991; Davis et al. 2010). In eukaryotic cells, about one‐third of proteins, including all integral membrane proteins, fold within the endoplasmic reticulum (Braakman and Hebert 2013). Interestingly, only two cyclophilins, CypB and CypC, are localized to this compartment. To determine whether the ER‐resident prolyl isomerase CypB is specifically adapted to the unique chemical environment of the ER, we carried out a detailed characterization of this protein.

As previously demonstrated in structural studies and predicted by the high sequence similarity (Davis et al. 2010; Kozlov et al. 2010; Mikol et al. 1994; Price et al. 1991), the secondary structure content of CypB closely resembles the canonical cyclophilin fold, and, similar to CypA, the protein remains structurally intact under physiological conditions. Cooperative urea‐induced unfolding transitions indicate that the catalytic domains of both CypA and CypB form thermodynamically stable, independent folding units. Furthermore, activity measurements using a protease‐free fluorescent peptide assay revealed that CypB exhibits high catalytic efficiency across multiple substrates, showing no clear preference for the residue preceding proline. This behavior mirrors the reported properties of CypA and data for CypD, supporting the idea that cyclophilin catalytic cores are highly conserved and substrate promiscuous (Schmidpeter et al. 2011; Zoldak et al. 2009). Notably, the same functional characteristics are observed under ER‐mimicking conditions, including oxidizing redox potentials and millimolar Ca^2+^ concentration, indicating that the PPIase activity of CypB is not specifically tuned to the unique chemical environment of the ER.

Together, these findings support the general model in which prolyl isomerases, especially those without additional chaperone domains, do not prevent protein aggregation by binding and protecting folding protein chains, but rather by reducing the lifetime of aggregation‐prone folding intermediates by catalyzing the intrinsically slow *cis*/*trans* isomerization of prolyl bonds (Schmidpeter and Schmid 2015a). This makes the existence of an N‐terminal hydrophobic helix even more intriguing. Confocal imaging of mammalian cells expressing full‐length CypB fused to mCherry confirms strong enrichment in the ER, likely due to insertion of the predicted N‐terminal transmembrane helix into the ER membrane. Deletion of the N‐terminal segment disrupts this localization, leading to a uniform distribution throughout the cell. This agrees with previous work (Price et al. 1991) and demonstrates that the N‐terminus is necessary for ER targeting. Together with the unchanged catalytic activity in different chemical environments, this suggests that the physiological specificity of CypB is achieved through spatial positioning rather than catalytic divergence. Such a mechanism aligns with sequence analyses of the cyclophilin family, which revealed that isoform diversity mainly stems from differences in targeting sequences and potential interaction surfaces outside the conserved catalytic domain (Davis et al. 2010).

Anchoring CypB in the ER membrane via the predicted N‐terminal transmembrane helix may be central to its role in the folding of secreted and membrane proteins. It positions a highly active PPIase near the entry point of nascent polypeptide chains, enabling CypB to assist in early‐stage folding. Furthermore, CypB has been shown to recruit other chaperones, such as Erp57 (a protein disulfide isomerase) and calnexin/calreticulin (lectin‐like chaperones), into multi‐chaperone complexes that integrate several functions to assist protein folding, including general chaperone functions, oxidative folding activity, and peptide bond isomerization (Kozlov et al. 2010; Kozlov and Gehring 2020). Anchoring CypB to the membrane via a transmembrane helix makes this enzyme ideally suited to keep multi‐functional chaperone complexes close to the membrane. Such multi‐chaperone complexes that combine general chaperone function, oxidoreductase activity, and prolyl isomerase activity, tethered to the ER membrane, can serve as critical protein folding complexes and assist in the folding of co‐translationally translocated proteins and membrane proteins. In cells, synergistic effects of different chaperones are likely crucial for productive folding of a broad substrate spectrum, which is supported by the observation that PPIases can increase the efficiency of other chaperones (Schonbrunner and Schmid 1992), and recent results have provided insights into folding condensates as an efficient way to bring together different folding‐helper activities (Leder et al. 2025). This may position CypB as a central component of the early secretory folding machinery. Our work can provide another example of how localization signals and membrane association serve as critical factors in maintaining proteostasis in eukaryotic cells (Kopp et al. 2017; Vermue et al. 2025). Further structural and proteomic approaches will be essential to determine whether selective recruitment into ER‐resident folding complexes contributes specifically to spatial organization within the ER lumen.

## 4 CONCLUSION

In summary, CypB combines a highly conserved catalytic PPIase domain with an N‐terminal localization signal that anchors the enzyme to the ER. These findings support the idea that compartmentalization and protein–protein interactions, rather than intrinsic catalytic tuning, define the physiological roles of individual cyclophilin isoforms. Our data highlight subcellular localization as a primary determinant of CypB PPIase function.

## 5 METHODS

### 5.1 Cloning and construct design

The genes for CypA and CypB were obtained from Addgene (#79039 and #36127, respectively) and cloned into a modified pET11a (amp^R^) vector that carries an N‐terminal His_6_‐SUMO purification tag using sequence‐ and ligation‐independent cloning (see Table S4 for primer sequences). The genes and their respective vectors were PCR‐amplified with Phusion High‐Fidelity DNA Polymerase (ThermoFisher Scientific), purified (Monarch PCR & DNA Cleanup Kit, NEB), and digested for 30 min with T4 Polymerase (NEB) in the absence of nucleotides. The reactions were immediately placed on ice, and dCTP was added to halt the exonuclease activity of T4 Polymerase. Genes and vectors were combined at a 5:1 ratio, supplemented with T4 Ligation Buffer and T4 DNA Ligase (NEB), and incubated for 30 min. The ligase was then heat‐inactivated (20 min at 65 °C), and DpnI (NEB) was added to digest any remaining template DNA. The ligation mixture was transformed into DH5α (NEB), plated on LB agar containing 100 μg/mL ampicillin, and incubated overnight at 37 °C to allow for colony growth. Individual colonies were picked, grown in 5 mL LB, and plasmids were purified (Monarch Spin Plasmid Miniprep Kit, NEB). Successful cloning was confirmed via Sanger sequencing and whole‐plasmid sequencing (Eurofins Genomics). The truncation of CypB (Δ28 CypB) for expression in bacteria was introduced via blunt‐end PCR. For expression in HEK293S GnTi^−^ cells (ATCC), CypB and Δ38 CypB were cloned into a modified BacMam vector with a C‐terminal mCherry‐His_8_ fusion tag following the same protocol (see Table S4 for primer sequences).

### 5.2 Protein expression and purification

Sequence‐verified plasmids for the different cyclophilins were transformed into *E. coli* BL21(DE3) (NEB) and plated on LB agar containing 100 μg/mL ampicillin. For protein expression, LB cultures (1–4 L, 100 μg/mL ampicillin) were grown at 37 °C (220 rpm) to an optical density of OD_600_ = 0.6, at which point protein expression was induced by the addition of IPTG (Isopropyl β‐D‐thiogalactoside, 1 mM final concentration). Cultures were further grown for 3 h (37 °C, 220 rpm). Cells were pelleted by centrifugation (10 min, 8 °C, 7000 *g*) and stored at −80 °C. To purify the desired PPIase, cells were thawed and resuspended in breaking buffer (50 mM Tris, 100 mM KCl, pH 7.8) supplemented with Lysozyme (Sigma Aldrich), DNase I (Sigma Aldrich), PMSF (phenylmethylsulfonyl fluoride, Roche), and complete Protease Inhibitor (Roche). Cells were broken by sonication, and insoluble particles and cell debris were removed by centrifugation (45 min, 4 °C, 37,000 *g*). The supernatant was loaded onto a 5 mL HiTrap Chelating column (Cytiva) charged with Ni^2+^ and pre‐equilibrated in buffer (20 mM HEPES, 100 mM KCl, 40 mM imidazole, pH 7.8). The column was washed with 15 CV of buffer, and the His_6_‐SUMO‐PPIase fusion protein was eluted in 15 mL of elution buffer (20 mM HEPES, 100 mM KCl, 300 mM imidazole, pH 7.8). To cleave off the purification tag, the eluted fusion protein was incubated overnight in the presence of the SUMO‐specific protease hSenp2 (Addgene # 71466) while dialyzing against 2 L of buffer (20 mM HEPES, 100 mM KCl, pH 7.8, 0.1 mM 2‐mercaptoethanol) at 4 °C. To remove the His_6_‐SUMO protein and any uncleaved PPIase fusion, the sample was loaded onto a 5 mL HiTrap Chelating column (Cytiva) charged with Ni^2+^ and pre‐equilibrated in buffer (20 mM HEPES, 100 mM KCl, pH 7.8). The flow‐through containing tag‐free PPIase was collected, concentrated (Amicon Ultra‐15, 3500 MWCO), and injected onto a HiLoad 16/600 Superdex 75 pg gel filtration column (Cytiva) equilibrated in buffer (20 mM HEPES, 100 mM KCl, pH 7.4) at room temperature. Protein‐containing fractions were pooled, concentrated (Amicon Ultra‐15, 3500 MWCO) to about 2 mL, and the final protein concentration was determined using a microspectrophotometer (DS‐11+, DeNovix) and extinction coefficients of 8730 M^−1^ cm^−1^ for CypA and 16,960 M^−1^ cm^−1^ for CypB.

CypD (UniProt P30405, residues 30–207, hereafter called CypD) was expressed from a pET11a with a C‐terminal GGSGSG‐His_6_ purification tag (Schmidpeter et al. 2020). Purification for this construct was essentially the same as for CypA and CypB. Since this construct does not have a cleavable tag, the protein was immediately concentrated after immobilized metal ion affinity chromatography (IMAC) and subjected to gel filtration.

The purity of the final protein samples was confirmed by SDS‐polyacrylamide gel electrophoresis (SDS‐PAGE) using 12% or 4–20% gradient Bis‐Tris gels (SurePAGE, GenScript) and defined molecular weight standards (PAGE‐MASTER Protein Standard Plus, or Broad Multi Color Pre‐Stained Protein Standard, GenScript).

### 5.3 High‐resolution mass spectrometry

For intact protein mass analysis of CypA and CypB purified from bacterial expression systems, electrospray ionization mass spectra (ESI‐MS) were acquired on a maXis plus quadrupole‐time‐of‐flight mass spectrometer (Bruker Daltonics) operating in positive‐ion mode. Samples were desalted by washing with 50 mM ammonium acetate using 3.5 kDa centrifugal spin filters. Working samples were prepared by diluting the desalted stock solutions to 10 μM in 50/49.9/0.1% methanol/water/acetic acid and introduced into the ESI source via a syringe pump at a constant flow rate of 3 μL/min. Key source parameters were: capillary voltage, 3500 V; nebulizer gas pressure, 0.4 bar; dry gas flow rate, 4.0 L/min; source temperature, 200 °C. Mass spectra were averaged over 1 min of scans collected at 1 scan per second in the range 50 ≤ *m*/*z* ≤ 3000. Mass spectra were processed using Compass Data Analysis software, version 4.3 (Bruker Daltonics).

For CypB expressed in HEK cells, a nanoLC‐MS consisting of a VanquishNeo nanoLC connected to an Orbitrap Exploris 480 mass spectrometer (ThermoFisher, San Jose, CA) was used for all analyses. 20 ng of the purified protein was loaded onto an Easy‐Spray MAbPAC RP column (150 μm × 150 mm, 4 μm, 1500 Å, 550 bar max, ThermoFisher Scientific) at 2 μL/min for intact protein mass analysis. Solvent A (0.1% formic acid in water) and B (80/20% acetonitrile/water containing 0.1% formic acid) were used to establish the 8 min gradient elution from 30 to 75% B. The Orbitrap Exploris 480 mass spectrometer was operated in positive‐ion mode over 150 ≤ *m*/*z* ≤ 2000, with a resolution of 120,000. Other important parameters were: ion source voltage, 1900 V; ion transfer tube temperature, 275 °C; RF lens, 70%; normalized AGC target, 100%; maximum injection time, 200 ms; ion energy, 15 V. For peptide mapping, purified CypB overexpressed in HEK cells was digested overnight with proteomics‐grade trypsin (Pierce™ Trypsin Protease, MS‐Grade, ThermoFisher Scientific) at a 1:50 enzyme:substrate ratio. Reversed‐phase liquid chromatography was performed using an Easy‐Spray PepMap Neo column (C18, 75 μm × 150 mm, 2 μm, 100 Å, 1500 bar max, ThermoFisher Scientific). The flow rate was maintained at 0.350 μL/min. Solvent A (0.1% formic acid in water) and B (80/20% acetonitrile/water containing 0.1% formic acid) were used to establish a 20 min gradient elution timetable: 2–40% B over 15 min, followed by 40–95% B in 5 min. The Orbitrap Exploris 480 mass spectrometer was operated in positive‐ion mode with EASY‐IC™ Lock Mass Correction applied at the start of each run and data‐dependent analysis. The instrument was operated with a full‐scan range of 150 ≤ *m*/*z* ≤ 2000 at a resolution of 60,000. The normalized automatic gain control was set to 300%. The precursor ion selection width was kept at 1.3 *m*/*z*, and peptide fragmentation was achieved by HCD (normalized collision energy 30%). Fragment ion scans were recorded at a resolution of 15,000, with the maximum injection time set to auto. Dynamic exclusion was enabled and set to 10 s. Mascot software (Matrix Science, Boston, MA) was used for protein identification and searched against the UniProt *Homo sapiens* (human) protein database.

### 5.4 Circular dichroism spectroscopy

Circular dichroism spectra were recorded on a J‐1100 CD spectrometer (Jasco) equipped with a Peltier element. A 10 μM protein solution (in 2 mM HEPES, 10 mM KCl, pH 7.4) was incubated at 15 °C in a quartz cuvette (1 mm pathlength) for 10 min. Eight spectra from 250 to 180 nm were recorded at 0.1 nm intervals, averaged, and corrected for the buffer signal. The mean residue molar ellipticity [Θ]_MRE_ was calculated according to Equation (1) for data presentation,

(1) $${\left[\theta \right]}_{\mathrm{MRE}}=\frac{100\cdot \theta_{\mathrm{obs}}}{l\cdot c\cdot N},$$

where Θ_obs_ is the observed ellipticity (mdeg), *l* is the path length (cm), *c* is the protein concentration (mol/L), and *N* is the number of amino acids.

### 5.5 Fluorescence spectra

Fluorescence spectra were recorded at 15 °C using an FP‐8350 fluorescence spectrophotometer (Jasco) equipped with an ETC‐115 Peltier element. 1 μM of protein was incubated for at least 30 min in 20 mM HEPES, 100 mM KCl, pH 7.4, ±6 M urea before measuring the fluorescence spectra from 295 to 450 nm (bandwidth: 5 nm) after excitation at 280 nm (bandwidth: 2 nm) with 200 nm/min, 0.1 s response, medium sensitivity. Protein spectra were corrected for the buffer fluorescence.

### 5.6 Equilibrium unfolding transitions

The urea‐induced unfolding transitions of CypA and CypB were measured at 15 °C using a FP‐8350 fluorescence spectrophotometer (Jasco) equipped with an ETC‐115 Peltier element. All samples were incubated at room temperature for at least 1 h before measuring the fluorescence of 1 μM PPIase (20 mM HEPES, 100 mM KCl, pH 7.4 and increasing concentrations of urea) at 350 nm (bandwidth: 5 nm) after excitation at 280 nm (bandwidth: 2 nm) in a 10 mm Macro Fluorescence Cuvette (QS 10 mm, Hellma) equilibrated to 15 °C under constant stirring (300 rpm). Samples without protein were recorded in the same way (1 M urea steps) to determine and correct for the buffer fluorescence during analysis. The urea concentration of each sample was calculated from the refractive index (Pace 1986). Buffer‐corrected fluorescence unfolding transitions were analyzed according to a two‐state model, describing the equilibrium between the fully folded (*N*) and unfolded (*U*) protein (N⇌U), using the linear extrapolation method (Equation (2)) (Santoro and Bolen 1988; Tanford 1970). The midpoint of the transition [urea]_M_ was calculated using the Gibb's free energy of unfolding Δ*G* and the cooperativity parameter *m*,

(2) $$y\left(D\right)=\frac{\left(y_{0}^{N}+m_{N}\cdot \left[D\right]\right)+\left(y_{0}^{U}+m_{U}\cdot \left[D\right]\right)\cdot e^{-\left(\frac{∆G}{\mathrm{RT}}+\frac{m\cdot \left[D\right]}{\mathrm{RT}}\right)}}{1+e^{-\left(\frac{∆G}{\mathrm{RT}}+\frac{m\cdot \left[D\right]}{\mathrm{RT}}\right)}},$$

where y(D) is the fluorescence signal as a function of the denaturant concentration, [D] is the urea concentration (M), $y_{N}^{0}$ and $y_{0}^{U}$ are the theoretical fluorescence values for native and unfolded protein in the absence of urea, *m*_*N*_ and *m*_U_ are the slopes of the baselines for native and unfolded protein (M^−1^), *m* is the cooperativity parameter (kJ mol^−1^ M^−1^), Δ*G* is the free energy change of unfolding (kJ mol^−1^), *R* is the universal gas constant (kJ mol^−1^ K^−1^), and *T* is the temperature (K).

### 5.7 Fluorescence‐based isomerization assay

Activity assays were performed at 15 °C using a FP‐8350 fluorescence spectrophotometer (Jasco) equipped with an ETC‐115 Peltier element. Peptides with the general formula Abz‐Ala‐Xaa‐Pro‐Phe‐pNA (Abz = amino benzoyl, Xaa = any amino acid, pNA = para‐Nitroanilide) were custom‐synthesized by GenScript. Peptides containing Ala, Glu, Lys, Leu, or Tyr before the proline were purchased with a purity of over 95% as determined by HPLC. Stock solutions of each peptide were prepared by dissolving the peptide in 0.55 M anhydrous LiCl/TFE (trifluoroethanol, Sigma Aldrich) to a final concentration of 750 μM. Peptide solutions were stored in a desiccator at 4 °C.

The peptide‐based activity assay (Zoldak et al. 2009) was performed under two conditions that mimic either the reducing environment of the cytosol (50 mM K_2_HPO_4_, 1 mM EDTA, pH 7.4, 1 mM dithiothreitol, DTT) or the oxidizing environment of the endoplasmic reticulum (ER) (20 mM HEPES, 100 mM KCl, 2 mM CaCl_2_, pH 7.4, 1 mM glutathione (GSH), 5 mM glutathione disulfide [GSSG]). 995 μL of buffer containing the desired concentration of PPIase was added to a 10 mm Macro Fluorescence Cuvette (QS 10 mm, Hellma) with a micro stir bar. The solution was equilibrated at 15 °C for 2 min. To initiate the peptidyl‐prolyl *cis*⇌*trans* re‐equilibration, 5 μL of the peptide stock solution (from anhydrous LiCl/TFE) was quickly added to the pre‐equilibrated aqueous PPIase solution in the cuvette to achieve a final peptide concentration of 3.75 μM while stirring continuously at 300 rpm. The fluorescence was recorded every second at 416 nm (bandwidth: 5 nm) following excitation at 316 nm (bandwidth: 2 nm), with a response time of 0.1 s and medium sensitivity.

Fluorescence time courses were analyzed using an exponential function. For some peptides, we observed a double‐exponential increase in fluorescence, with the lower rate unaffected by PPIases and the faster rate increasing linearly with PPIase concentration. This could be due to different peptide synthesis strategies compared to previous studies (Zoldak et al. 2009), which may leave residual salts in the final, lyophilized peptide (such as trifluoroacetic acid). Another possible explanation is that other contaminants that HPLC cannot remove may interfere with catalysis (purity ≥95%). We performed at least four independent repeats for each PPIase concentration with enzyme from two independent purifications, averaged the rates, and determined the standard deviation (SD). The averaged rate constants sensitive to PPIases were plotted as a function of the PPIase concentration. Linear regression analysis was performed on these plots to determine the specificity constant (*k*_cat_/*K*_M_) for each condition, as described in Equation (3),

(3) $$k_{\mathrm{app}}=k_{0}+\left[E\right]\cdot \frac{k_{\mathrm{cat}}}{K_{M}},$$

where *k*_app_ is the measured rate constant (s^−1^), *k*_0_ is the rate of the uncatalyzed reaction (s^−1^), and [E] is the enzyme concentration.

### 5.8 Mammalian cell culture and virus production

Full‐length and truncated (Δ38, residues 39–216) human CypB (Uniprot: P23284) were cloned into a modified pEG BacMam vector (Goehring et al. 2014) containing a C‐terminal mCherry‐His_10_ tag preceded by a 3C protease cleavage site. Plasmids were transformed into DH10Bac cells (NEB) to generate Bacmid DNA, then transfected into adherent *Spodoptera frugiperda* Sf9 cells (Gibco) in Sf‐900 III SFM media (supplemented with 100 units/mL penicillin and 100 μg/mL streptomycin) using cellfectin II (Gibco). All virus passages were harvested by pelleting the insect cells (10 min, 20 °C, 2000 *g*) and passing the supernatant through a 0.22 μm sterile filter. The virus was then stored in 2% FBS at 4 °C, protected from light, until use.

Adherent HEK 293S GnTi^−^ cells (ATCC) were grown to 80% confluence in DMEM (10% FBS, 100 units/mL penicillin, 100 μg/mL streptomycin, 2 mM L‐glutamine, 5% CO_2_) and co‐transduced by infection with P2 virus (10%) and transfection with CellLight ER‐GFP (Invitrogen, 12.5 particles per cell [PPC]). 16 h later, Na‐butyrate (5 mM) was added, and the temperature was decreased to 30 °C. At 24 h post‐induction, cells were diluted 1:1 with DMEM and transferred to a clean well with a Poly‐L‐Lysine‐treated coverslip (Neuvitro), then allowed to adhere overnight at 30 °C. Cells were fixed 48 h post‐induction with fresh formaldehyde (3.7% in PBS, 15 min), stained with the dsDNA dye Hoechst (Invitrogen: 33342, 1 μg/mL in PBS, 20 min), and mounted on a microscope slide with 90% glycerol in PBS. Slides were allowed to dry at room temperature overnight, then sealed with clear nail polish and stored at 4 °C, protected from light, until use.

### 5.9 Confocal microscopy

Confocal imaging was performed at the UTSA Cell Analysis Core using a Zeiss 710 NLO 2P System. All images were taken using a 100x/1.40 oil lens, monitoring Hoechst at 410–587 nm (405 nm laser), EGFP at 495–592 nm (488 nm laser), and mCherry at 578–696 nm (561 nm laser). Z‐stacks were automatically acquired for each cell, and 3D projections were assembled using the Imaris software v10 (Oxford Instruments). For each image, a contrast/histogram stretch was performed, and the gamma values for the Hoechst, EGFP, and mCherry signals were increased to 1.5 (Cromey 2010).

## AUTHOR CONTRIBUTIONS

**Wendell P. Griffith:** Investigation; formal analysis; data curation; validation; writing – review and editing. **Philipp A. M. Schmidpeter:** Conceptualization; investigation; formal analysis; validation; data curation; supervision; funding acquisition; writing – original draft; project administration; visualization; writing – review and editing. **Gavin A. Grever:** Investigation; validation; writing – review and editing. **Thomas C. Yost:** Investigation; formal analysis; data curation; validation; writing – review and editing; visualization. **Sarah C. DeVoe:** Investigation; formal analysis; data curation; validation; visualization; funding acquisition; writing – review and editing. **Robert D. Latvala:** Investigation; formal analysis; writing – review and editing. **Melissa Fernandez Ayala:** Investigation. **Ashley J. Newton:** Investigation; formal analysis; data curation; validation; visualization; writing – review and editing.

## CONFLICT OF INTEREST STATEMENT

The authors declare no conflicts of interest.

## Supporting information


**Figure S1.** Sequence alignment of human cyclophilins. Multiple sequence alignment generated using CLUSTAL O (1.2.4) with the UniProt entries for human cyclophilins: P62937 (CypA), P23284 (CypB), P45877 (CypC), P30405 (CypD), Q9UNP9 (CypE), Q13427 (CypG), and O43447 (CypH). Identical residues across all isoforms are highlighted in dark yellow, and similar residues in light blue. Active site residues are marked by triangles (▼), and horizontal arrows (→ ←) denote the domain limitations of the cyclophilin catalytic core. These data supplement Figure 1.
**Figure S2.** Expression and purification of cyclophilins. (a) AlphaFold model of CypB (AF‐P23284‐F1‐v4) showing the predicted N‐terminal helix. Model is colored by model confidence (pLDDT) with blue = very high (pLDDT > 90), yellow = low (pLDDT > 50), and red = very low (pLDDT < 50). Positions at which the protein was truncated for this work are indicated. (b) Prediction of transmembrane helices using TMHMM‐2.0 (Krogh et al. 2001) with full‐length CypB (UniProt: P23284) as input. (c–e) Gel filtration (HiLoad 16/600 Superdex 75 pg) with SDS‐PAGE analysis of the final purified protein samples for CypA in (c), CypB in (d), and CypD in (e). MW indicates molecular weight markers with PAGE‐MASTER Protein Standard Plus (GenScript) used in (c, d), and Broad Multi Color Pre‐Stained Protein Standard (GenScript) in (e). (f, g) High‐resolution mass spectrometry results confirming the identity of purified CypA in (f), and CypB in (g), by comparing the experimentally determined mass (expt) of the intact proteins with the calculated mass (calc) based on the amino acid sequence. These data support the results presented in Figures 1–4.
**Figure S3.** Catalytic activity of cyclophilins under reducing conditions. (a–d) Apparent rate of fluorescence change (which reports on prolyl isomerization) as a function of the PPIase concentration for different peptide substrates of the general formula Abz‐Ala‐Xaa‐Pro‐Phe‐pNA (Abz = amino benzoyl, Xaa = any amino acid, pNA = para‐Nitroanilide). The exact peptide sequence is indicated in the top‐left corner of each panel. Data points are mean ± SD from at least three repeats. Lines are linear fits over averaged data points to determine *k*_cat_/*K*_M_ according to Equation (3). Data are presented for CypA (in (a)), for CypB (in (b)), for CypD (in (c)), and for SlyD (in (d)). All experiments were performed at 15°C in 50 mM K_2_HPO_4_, 1 mM EDTA, pH 7.4, 1 mM dithiothreitol (DTT). These data correspond to Figure 3.
**Figure S4.** Activity of cyclophilins under ER‐mimicking conditions. (a, b) Apparent rate of fluorescence change (which reports on prolyl isomerization) as a function of the PPIase concentration for different peptide substrates of the general formula Abz‐Ala‐Xaa‐Pro‐Phe‐pNA (Abz = amino benzoyl, Xaa = any amino acid, pNA = para‐Nitroanilide). The exact peptide sequence is indicated in the top‐left corner of each panel. Data points are mean ± SD from at least three repeats. Lines are linear fits over averaged data points to determine *k*_cat_/*K*_M_ according to Equation (3). Data are presented for CypA (in (a)) and for CypB (in (b)). All experiments were performed at 15 °C in 20 mM Hepes, 100 mM KCl, 2 mM CaCl_2_, pH 7.4, 1 mM glutathione (GSH), 5 mM glutathione disulfide (GSSG). The presented data correspond to Figure 4.
**Figure S5.** Mass spectrometry of CypB expressed in HEK cells. (a) High‐resolution mass spectrometry results showing that full‐length CypB is partially degraded during purification, as indicated by the difference between the experimentally determined mass (expt) and the calculated mass (calc) of the protein based on the amino acid sequence. (b) Mapping of mass spectrometry‐identified peptides (in red) showing the sequence coverage for full‐length CypB (after cleavage of the mCherry‐His_10_ tag by 3C protease).
**Figure S6.** Confocal microscopy of CypB expressed in HEK cells. (a, b) Additional confocal microscopy images of HEK cells captured in the center slice of the cell. Columns are (from left to right): brightfield, mCherry (CypB), GFP (ER), Hoechst (dsDNA), and merge of the three fluorescence channels. The CypB variants expressed are human full‐length CypB (in (a)), and human CypB with the first 38 amino acids deleted (Δ38 CypB, in (b)). The scale bar in all images is 10 μm. These data correspond to Figure 5.
**Table S1.** This summarizes the numerical stability data for CypA and CypB. The values presented are averages from three independent experiments ± SD. These data correspond to Figure 2c,d.
**Table S2.** This summarizes *k*_cat_/*K*_M_ values for CypA, CypB, and CypD under reducing conditions (mimicking the cytosolic environment). These data correspond to Figure 3.
**Table S3.** This summarizes *k*_cat_/*K*_M_ values for CypA and CypB under oxidizing conditions (mimicking the chemical environment of the ER). These data correspond to Figure 4.
**Table S4.** This summarizes the primer sequences used to clone the constructs in this study.

## Data Availability

All data needed to evaluate the conclusions in the paper are present in the paper and/or the Supporting Information. Raw data supporting the findings of this manuscript are available from the corresponding author upon request.
