# Supplementary material for: Biophysical characterization of Cyclophilin B reveals membrane localization as its primary functional determinant as a prolyl isomerase
Source: Protein Sci. 2026 Apr 16;35(5):e70579. doi: 10.1002/pro.70579 (PMC13084196; doi:10.1002/pro.70579)
Supplement: Supplementary file 1 — Figure S1. Sequence alignment of human cyclophilins. Multiple sequence alignment generated using CLUSTAL O (1.2.4) with the UniProt entries for human cyclophilins: P62937 (CypA), P23284 (CypB), P45877 (CypC), P30405 (CypD), Q9UNP9 (CypE), Q13427 (CypG), and O43447 (CypH). Identical residues across all isoforms are highlighted in dark yellow, and similar residues in light blue. Active site residues are marked by triangles (▼), and horizontal arrows (→ ←) denote the domain limitations of the cyclophilin catalytic core. These data supplement Figure 1. Figure S2. Expression and purification of cyclophilins. (a) AlphaFold model of CypB (AF‐P23284‐F1‐v4) showing the predicted N‐terminal helix. Model is colored by model confidence (pLDDT) with blue = very high (pLDDT > 90), yellow = low (pLDDT > 50), and red = very low (pLDDT < 50). Positions at which the protein was truncated for this work are indicated. (b) Prediction of transmembrane helices using TMHMM‐2.0 (Krogh et al. 2001) with full‐length CypB (UniProt: P23284) as input. (c–e) Gel filtration (HiLoad 16/600 Superdex 75 pg) with SDS‐PAGE analysis of the final purified protein samples for CypA in (c), CypB in (d), and CypD in (e). MW indicates molecular weight markers with PAGE‐MASTER Protein Standard Plus (GenScript) used in (c, d), and Broad Multi Color Pre‐Stained Protein Standard (GenScript) in (e). (f, g) High‐resolution mass spectrometry results confirming the identity of purified CypA in (f), and CypB in (g), by comparing the experimentally determined mass (expt) of the intact proteins with the calculated mass (calc) based on the amino acid sequence. These data support the results presented in Figures 1–4. Figure S3. Catalytic activity of cyclophilins under reducing conditions. (a–d) Apparent rate of fluorescence change (which reports on prolyl isomerization) as a function of the PPIase concentration for different peptide substrates of the general formula Abz‐Ala‐Xaa‐Pro‐Phe‐pNA (Abz = amino benzoyl, Xaa = any [file PRO-35-e70579-s001.pdf]

Biophysical characterization of Cyclophilin B reveals membrane localization as its primary functional determinant as a prolyl isomerase

Sarah C. DeVoe*, Thomas C. Yost*, Ashley J. Newton, Gavin A. Grever, Melissa Fernandez Ayala, Wendell P. Griffith, Robert D. Latvala, and Philipp A.M. Schmidpeter#

Department of Chemistry, The University of Texas at San Antonio, One UTSA Circle, San Antonio, TX 78249, USA

* equal contribution

# correspondence to: philipp.schmidpeter@utsa.edu

Supplementary Information

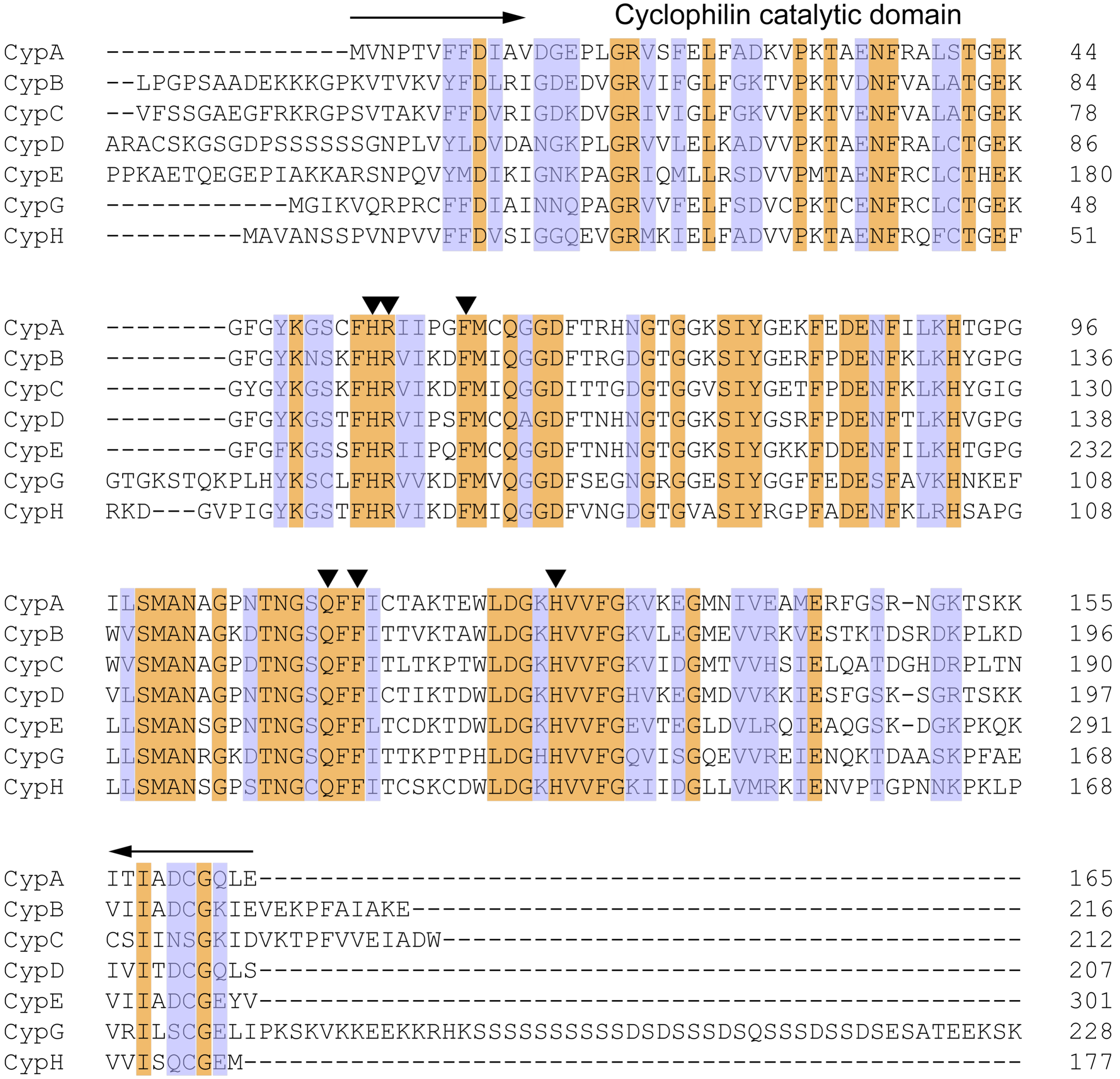

Supplementary Figure 1: Sequence alignment of human cyclophilins
Multiple sequence alignment generated using CLUSTAL O (1.2.4) with the UniProt entries for human cyclophilins: P62937 (CypA), P23284 (CypB), P45877 (CypC), P30405 (CypD), Q9UNP9 (CypE), Q13427 (CypG), and O43447 (CypH). Identical residues across all isoforms are highlighted in dark yellow, and similar residues in light blue. Active site residues are marked by triangles (▼), and horizontal arrows (→ ←) denote the domain limitations of the cyclophilin catalytic core. These data supplement Figure 1.

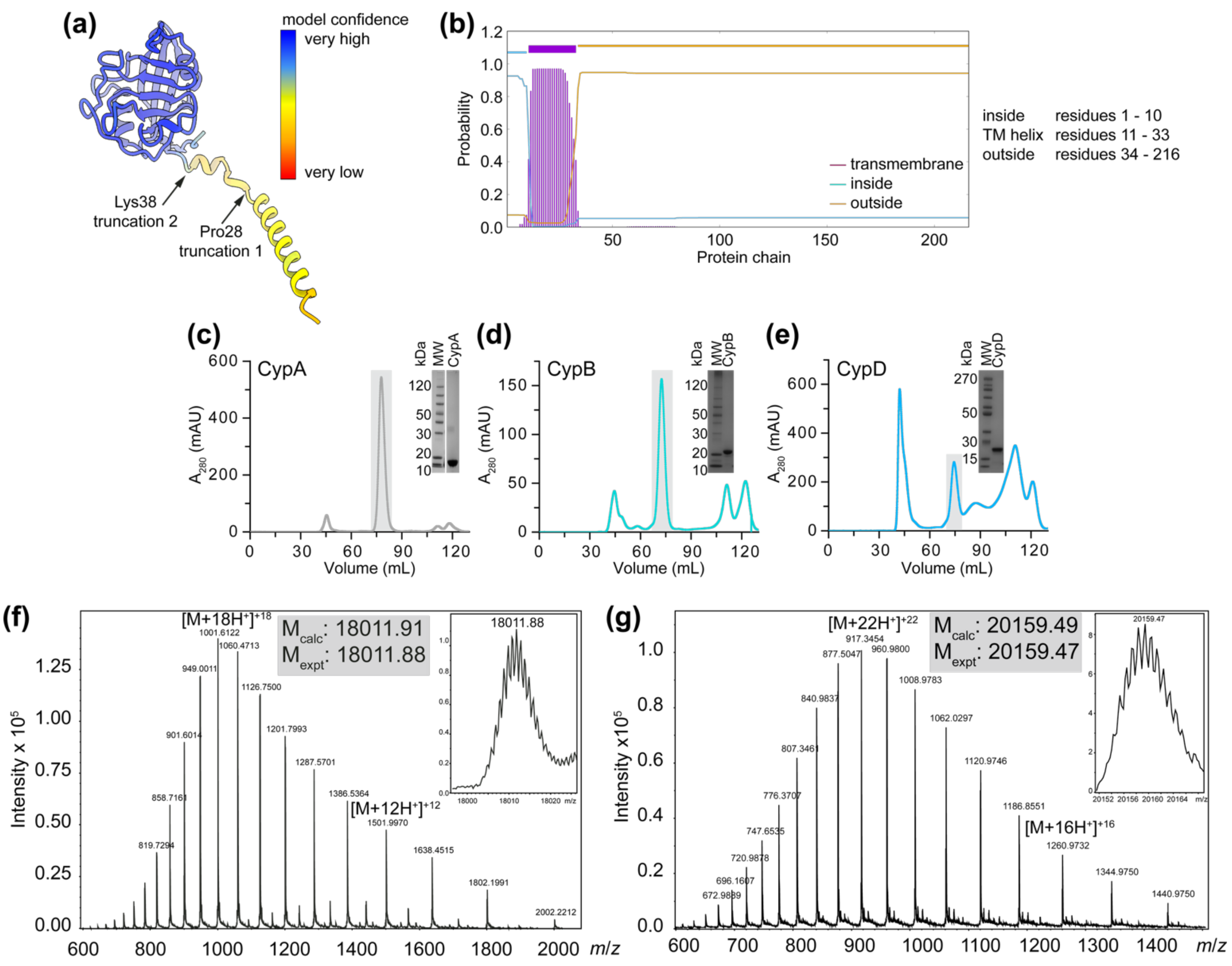

Supplementary Figure 2: Expression and purification of cyclophilins

a) AlphaFold model of CypB (AF-P23284-F1-v4) showing the predicted N-terminal helix. Model is colored by model confidence (pLDDT) with blue = very high (pLDDT > 90), yellow = low (pLDDT > 50), and red = very low (pLDDT < 50). Positions at which the protein was truncated for this work are indicated. **b)** Prediction of transmembrane helices using TMHMM – 2.0 (Krogh, Larsson et al. 2001) with full-length CypB (UniProt: P23284) as input. **c)**, **d)**, and **e)** Gel filtration (HiLoad 16/600 Superdex 75 pg) with SDS-PAGE analysis of the final purified protein samples for CypA in (c), CypB in (d), and CypD in (e). MW indicates molecular weight markers with PAGE-MASTER Protein Standard Plus (GenScript) used in (c) and (d), and Broad Multi Color Pre-Stained Protein Standard (GenScript) in (e). **f)** and **g)** High-resolution mass spectrometry results confirming the identity of purified CypA in (f), and CypB in (g), by comparing the experimentally determined mass (expt) of the intact proteins with the calculated mass (calc) based on the amino acid sequence. These data support the results presented in Figures 1 – 4.

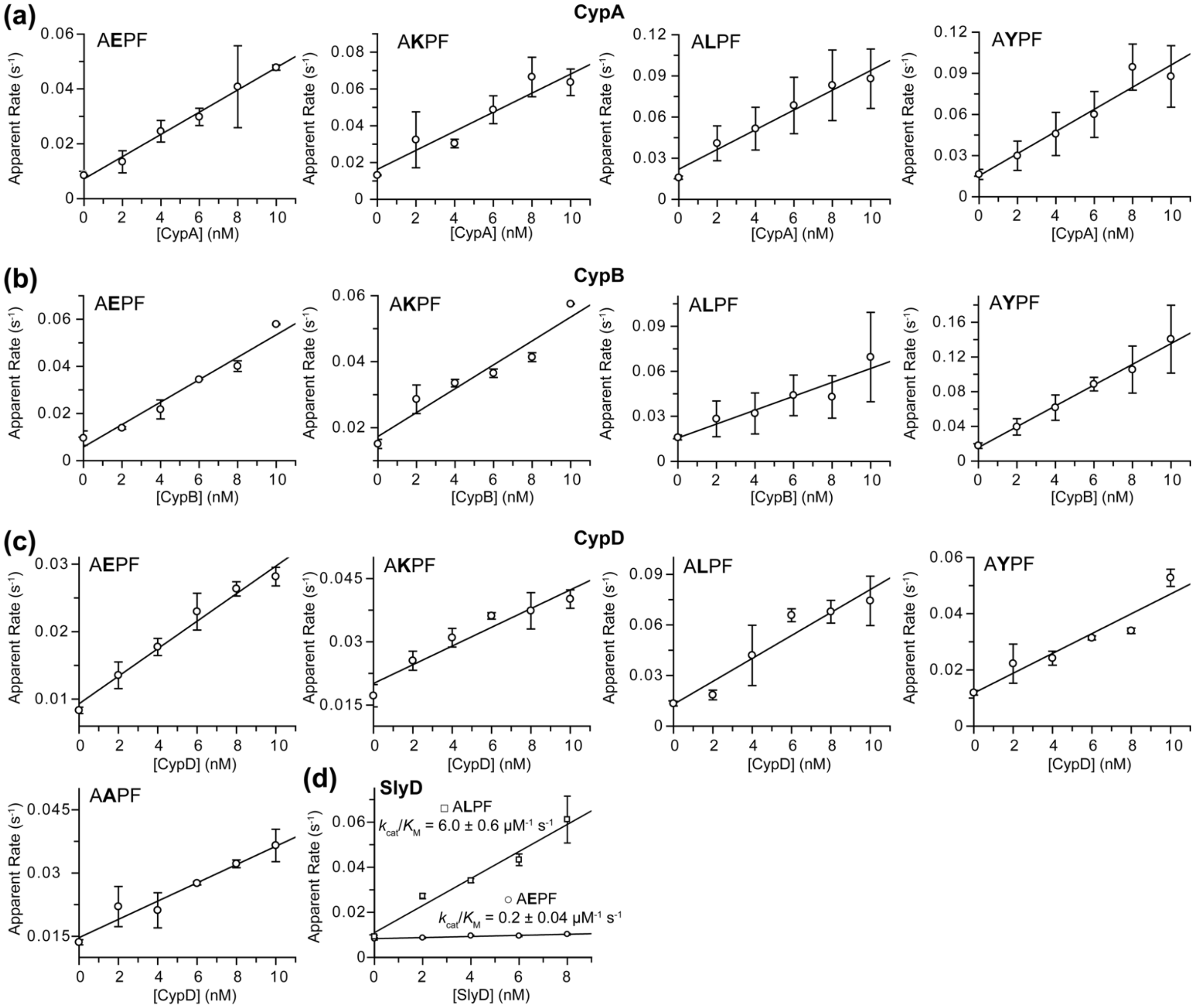

Supplementary Figure 3: Catalytic activity of cyclophilins under reducing conditions

a), **b)**, **c),** and **d)** Apparent rate of fluorescence change (which reports on prolyl isomerization) as a function of the PPIase concentration for different peptide substrates of the general formula Abz-Ala-Xaa-Pro-Phe-pNA (Abz = amino benzoyl, Xaa = any amino acid, pNA = para-Nitroanilide). The exact peptide sequence is indicated in the top-left corner of each panel. Data points are mean ± S.D. from at least three repeats. Lines are linear fits over averaged data points to determine k_{cat}/K_M according to Eq. 3. Data are presented for CypA (in (a)), for CypB (in (b)), for CypD (in (c)), and for SlyD (in (d)). All experiments were performed at 15 °C in 50 mM K_2HPO_4, 1 mM EDTA, pH 7.4, 1 mM dithiothreitol (DTT). These data correspond to Figure 3.

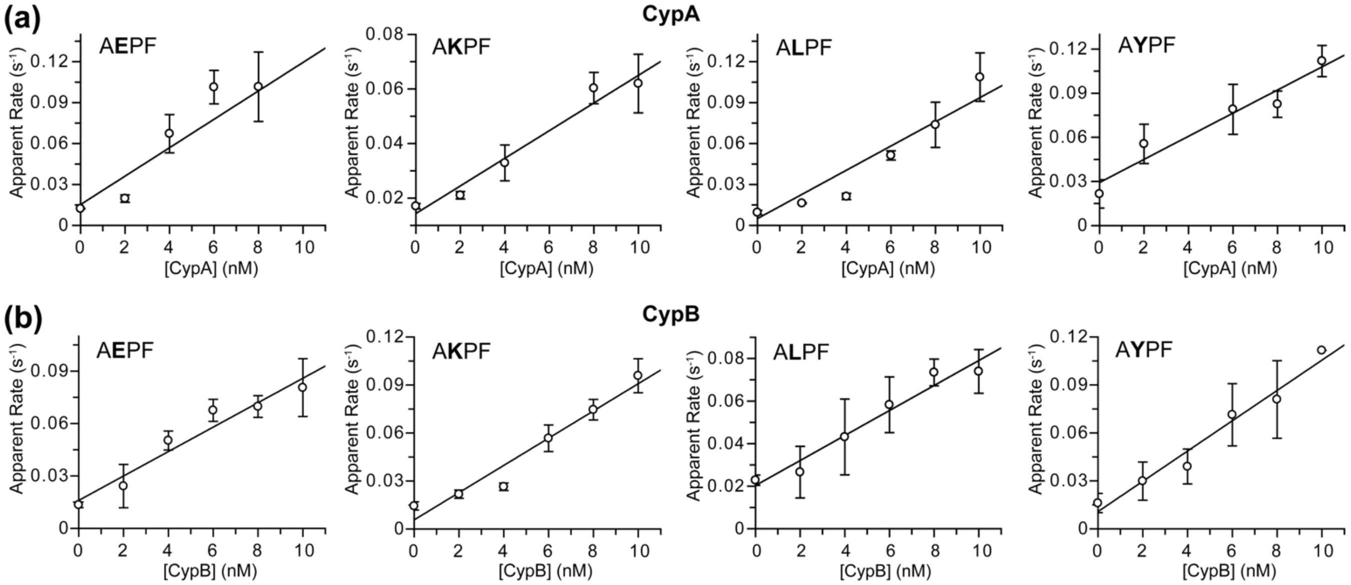

Supplementary Figure 4: Activity of cyclophilins under ER-mimicking conditions
a) and **b)** Apparent rate of fluorescence change (which reports on prolyl isomerization) as a function of the PPIase concentration for different peptide substrates of the general formula Abz-Ala-Xaa-Pro-Phe-pNA (Abz = amino benzoyl, Xaa = any amino acid, pNA = para-Nitroanilide). The exact peptide sequence is indicated in the top-left corner of each panel. Data points are mean ± S.D. from at least three repeats. Lines are linear fits over averaged data points to determine k_{cat}/K_M according to Eq. 3. Data are presented for CypA (in (a)) and for CypB (in (b)). All experiments were performed at 15 °C in 20 mM Hepes, 100 mM KCl, 2 mM $CaCl_2$, pH 7.4, 1 mM glutathione (GSH), 5 mM glutathione disulfide (GSSG). The presented data correspond to Figure 4.

(a)

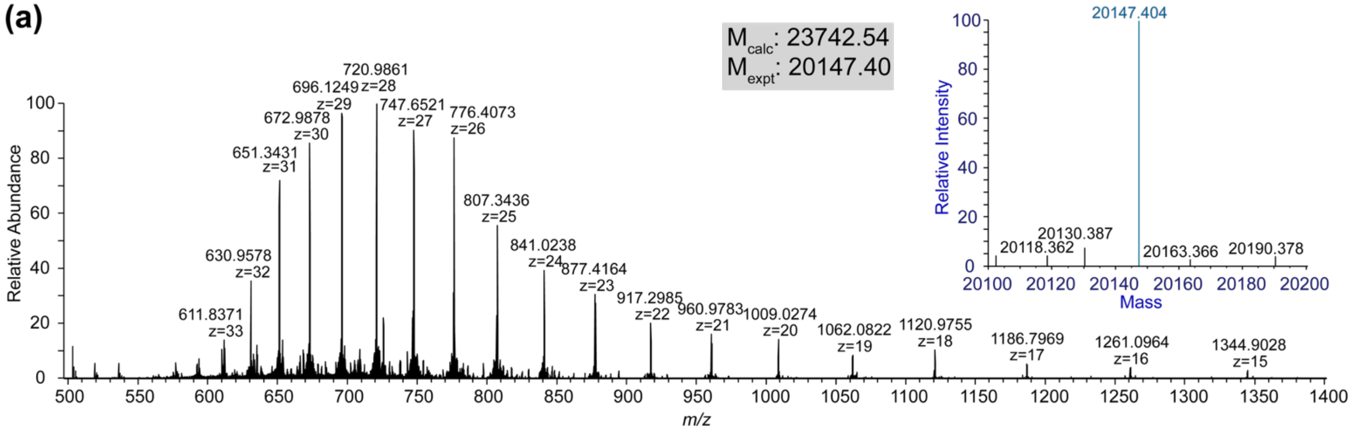

(b)

1 MLRLSERNMK VLLAAALIAG SVFFLLLPGP SAADEKKGP KVTVKVYFDL

51 RIGDEDVGRV IFGLFGKTVP KTVDNFVALA TGEKGFGYKN SKFHRVIKDF

101 MIQGGDFTRG DGTGGKSIYG ERFPDENFKL KHYGPGWVSM ANAGKDTNGS

151 QFFITTVKTA WLDGKHVVFG KVLEGMEVVR KVESTKTDSR DKPLKDVIIA

201 DCGKIEVEKP FAIAKE

Supplementary Figure 5: Mass spectrometry of CypB expressed in HEK cells

a) High-resolution mass spectrometry results showing that full-length CypB is partially degraded during purification, as indicated by the difference between the experimentally determined mass (expt) and the calculated mass (calc) of the protein based on the amino acid sequence. **b)** Mapping of mass spectrometry-identified peptides (in red) showing the sequence coverage for full-length CypB (after cleavage of the mCherry-His_{10} tag by 3C protease).

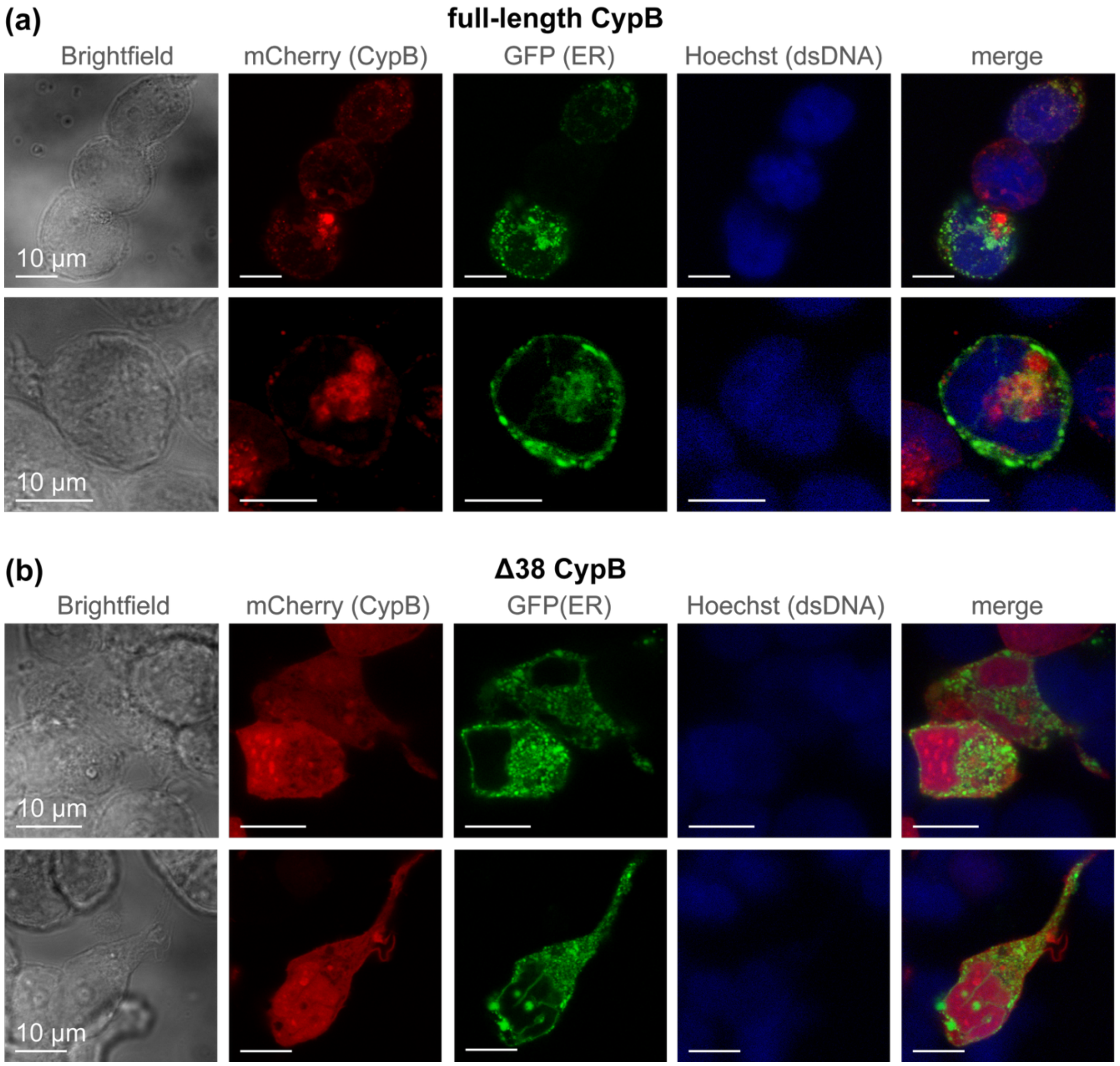

Supplementary Figure 6: Confocal microscopy of CypB expressed in HEK cells
a) and **b)** Additional confocal microscopy images of HEK cells captured in the center slice of the cell. Columns are (from left to right): brightfield, mCherry (CypB), GFP (ER), Hoechst (dsDNA), and merge of the three fluorescence channels. The CypB variants expressed are human full-length CypB (in (a)), and human CypB with the first 38 amino acids deleted (Δ38 CypB, in (b)). The scale bar in all images is 10 μm. These data correspond to Figure 5.

Supplementary Table 1

	[Urea] (M)	m (kJ mol^{-1} M^{-1})	$\Delta G^{15\,°C}$ (kJ mol^{-1})
**CypA**	4.56 ± 0.31	3.7 ± 0.7	17.0 ± 3.6
**CypB**	3.58 ± 0.06	15.5 ± 4.3	55.4 ± 1.5

Supplementary Table 1 summarizes the numerical stability data for CypA and CypB. The values presented are averages from three independent experiments ± S.D. These data correspond to Figure 2c and d.

Supplementary Table 2

	k_{cat}/K_M (M^{-1} s^{-1}) x 10^3		
Tetra-peptide	**CypA**	**CypB**	**CypD**
A**A**PF	1500 ± 100	2200 ± 200	2200 ± 200
A**E**PF	4100 ± 200	4800 ± 500	2000 ± 100
A**K**PF	5200 ± 700	3600 ± 500	2200 ± 300
A**L**PF	7200 ± 700	3100 ± 600	6800 ± 1000
A**Y**PF	7800 ± 130	12000 ± 500	3500 ± 600

Supplementary Table 2 summarizes k_{cat}/K_M values for CypA, CypB and CypD under reducing conditions (mimicking the cytosolic environment). These data correspond to Figure 3.

Supplementary Table 3

	k_{cat}/K_M (M^{-1} s^{-1}) x 10^3	
Tetra-peptide	**CypA**	**CypB**
A**A**PF	8100 ± 1000	6000 ± 1000
A**E**PF	10400 ± 200	7000 ± 900
A**K**PF	5100 ± 500	8500 ± 1000
A**L**PF	10000 ± 1400	5900 ± 600
A**Y**PF	7900 ± 1000	9500 ± 900

Supplementary Table 3 summarizes k_{cat}/K_M values for CypA and CypB under oxidizing conditions (mimicking the chemical environment of the ER). These data correspond to Figure 4.

Supplementary Table 4

Primer name	Primer Sequence
CypA_gene_fwd	5′- TCA CCG CGA ACA GAT TGG AGG TAT GGT CAA CCC CAC CGT GTT CT -3′
CypA_gene_rev	5′- GGC TTT GTT AGC AGC CGG ATC CTT ATT CGA GTT GTC CAC AGT CA -3′
CypA_vec_fwd	5′- TGA CTG TGG ACA ACT CGA ATA AGG ATC CGG CTG CTA ACA AAG CC -3′
CypA_vec_rev	5′- AGA ACA CGG TGG GGT TGA CCA TAC CTC CAA TCT GTT CGC GGT GA -3′
CypB_gene_fwd	5′- TCA CCG CGA ACA GAT TGG AGG TAT GCT GCG CCT CTC CGA ACG CA - 3′
CypB_gene_rev	5′- AGC TTC CTT TCG GGC TTT GTT ACT AAA AGG GCT TCT CCA CCT CG -3′
CypB_vec_fwd	5′- CGA GGT GGA GAA GCC CTT TTA GTA ACA AAG CCC GAA AGG AAG CT -3′
CypB_vec_rev	5′- TGC GTT CGG AGA GGC GCA GCA TAC CTC CAA TCT GTT CGC GGT GA -3′
CypB_Δ28_fwd	5′- GGA CCT TCT GCG GCC GAT GAG -3′
CypB_Δ28_rev	5′- ACC TCC AAT CTG TTC GCG GTG -3′
Bac_CypB_gene_fwd	5′- AGC GCG CTA GCC TCG AGC CAC CAT GCT GCG CCT CTC CGA ACG CA -3′
Bac_CypB_gene_rev	5′- GAC CCT GGA ACA GAA CTT CCA GAA AGG GCT TCT CCA CCT CGA TC -3′
Bac_CypB_vec_fwd	5′- GAT CGA GGT GGA GAA GCC CTT TCT GGA AGT TCT GTT CCA GGG TC -3′
Bac_CypB_vec_rev	5′- TGC GTT CGG AGA GGC GCA GCA TGG TGG CTC GAG GCT AGC GCG CT -3′
Bac_CypB_Δ38_gene_fwd	5′- AGC GCG CTA GCC TCG AGC CAC CAT GGG GCC CAA AGT CAC CGT CA -3′
Bac_CypB_Δ38_gene_rev	5′- GAC CCT GGA ACA GAA CTT CCA GAA AGG GCT TCT CCA CCT CGA TC -3′
Bac_CypB_Δ38_vec_fwd	5′- GAT CGA GGT GGA GAA GCC CTT TCT GGA AGT TCT GTT CCA GGG TC -3′
Bac_CypB_Δ38_vec_rev	5′- TGA CGG TGA CTT TGG GCC CCA TGG TGG CTC GAG GCT AGC GCG CT -3′

Supplementary Table 4 summarizes the primer sequences used to clone the constructs in this study.

References

Krogh, A., B. Larsson, G. von Heijne and E. L. Sonnhammer (2001). "Predicting transmembrane protein topology with a hidden Markov model: application to complete genomes." J Mol Biol **305**(3): 567-580.
